# Simple bone cyst: A case report

**DOI:** 10.4317/jced.57769

**Published:** 2021-02-01

**Authors:** Brais Pérez-Iglesias, Jesús Sandoval-Gutiérrez, Cayetana García-Freire, Alba Sánchez-Torres, Cosme Gay-Escoda

**Affiliations:** 1 Dentistry student. School of Medicine and Health Sciences, University of Barcelona, Barcelona, Spain; 2 MD, DDS, OMFS. University Complutense of Madrid. Private practice, Vigo, Spain; 3 DDS. University of Santiago de Compostela. Private practice, Vigo, Spain; 4 DDS, MS, Master of Oral Surgery and Implantology. Associate Professor of Oral Surgery and Professor of the Master’s degree program in Oral Surgery and Implantology, School of Medicine and Health Sciences, University of Barcelona. Researcher at the IDIBELL institute. Barcelona, Spain; 5 MD, DDS, MS, PhD, EBOS, OMFS. Chairman and Professor of Oral and Maxillofacial Surgery, School of Medicine and Health Sciences, University of Barcelona. Director of the Master Degree Program in Oral Surgery and Implantology (EHFRE International University/FUCSO). Coordinator/Researcher of the IDIBELL Institute. Head of the Department of Oral Surgery, Implantology and Maxillofacial Surgery, Teknon Medical Center. Barcelona, Spain

## Abstract

**Background:**

Simple bone cysts (SBC) are intraosseous cysts devoid of an epithelial lining, asymptomatic and appearing in the jaw. In general, SBCs are discovered incidentally and tooth displacement or pathological fractures are very unusual.

**Material and Methods:**

This study reports a 16 years old man that presented an asymtomatic radiolucent unilocular lesion in the right ascending ramus. Differencial diagnosis included odontogenic keratocyst and dentigerous cyst. One surgical intervention was performed and consisted in the curettage of the bone walls. The lesion and some small samples of the bone wall were sent for the anatomopathological study.

**Results:**

The anatomopathological exam confirmed the diagnosis of simple bone cyst. There was no evidence of recurrence after 6 months of follow-up and bone regeneration was almost complete.

**Conclusions:**

Curettage is the technique of choice for SBC treatment. Control visits are necessary to check the absence of postoperative complications and bone regeneration.

** Key words:**Simple bone cyst, traumatic bone cyst, intraosseous cyst, curettage.

## Introduction

Simple bone cyst (SBC) is an intraosseous cyst devoid of an epithelial lining, either empty or filled with serous or blood fluid, with a prevalence less than 1% (1,2). This lesion is included in the group of bone-related lesions, along with the aneurysmal bone cyst, ossifying fibroma, fibrous dysplasia, bone dysplasia, central giant cell granuloma, and cherubism (3,4). Moreover, it can also be referred to as traumatic bone cyst, hemorrhagic bone cyst, hemorrhagic cyst, unicameral bone cyst, idiopathic bone cyst. The new WHO classification released in 2017 defines the SBC as an intraosseus pseudocysct devoid of an ephitelial lining, either or filled with serous or sanguinous fluid. There are no differences between the classification of 2005 (3) and the new classification of 2017 (1).

SBCs appear almost exclusively in the jaws, specifically anterior areas, and are predominant in women (1.8:1) and young patients (5,6). Radiologically, they appear as a radiolucent image with irregular or scalloped but well-defined margins (4) and clinically, they are usually asymptomatic although some cases have reported mild pain (1,4).

The diagnosis of SBC is often casual and radiological. Tooth displacement or pathological fractures are very unusual, and a history of trauma is rarely reported (1). Harnet *et al.* (5) described three theories on the formation of SBC: 1) local abnormality during bone growth due to an abnormality in cell differentiation during the jaw ossification and growth with local environmental factors that induce mechanical restrictions during osteogenesis and angiogenesis; 2) SBC as part of a tumor process or a central giant cell granuloma after surgery (7); and 3) the lesion is caused by a low intensity trauma, based on the appearance of intramedullary hemorrhage followed by hematoma after insufficient trauma to fracture healthy bone.

The treatment of choice is the surgical approach to confirm the presumptive diagnosis. In the 90.5% of cases, the cavities appear empty. A simple curettage of the bony walls may be done as it favors progressive bone regeneration. Although this lesion has a low recurrence rate, clinical and radiological follow-ups after surgery are indicated in order to observe bone regeneration (4).

The objective of this case report was to describe the clinical and radiological characteristics of a simple bone cyst close to a semierupted lower third molar.

## Case Report

**Figure 1:**
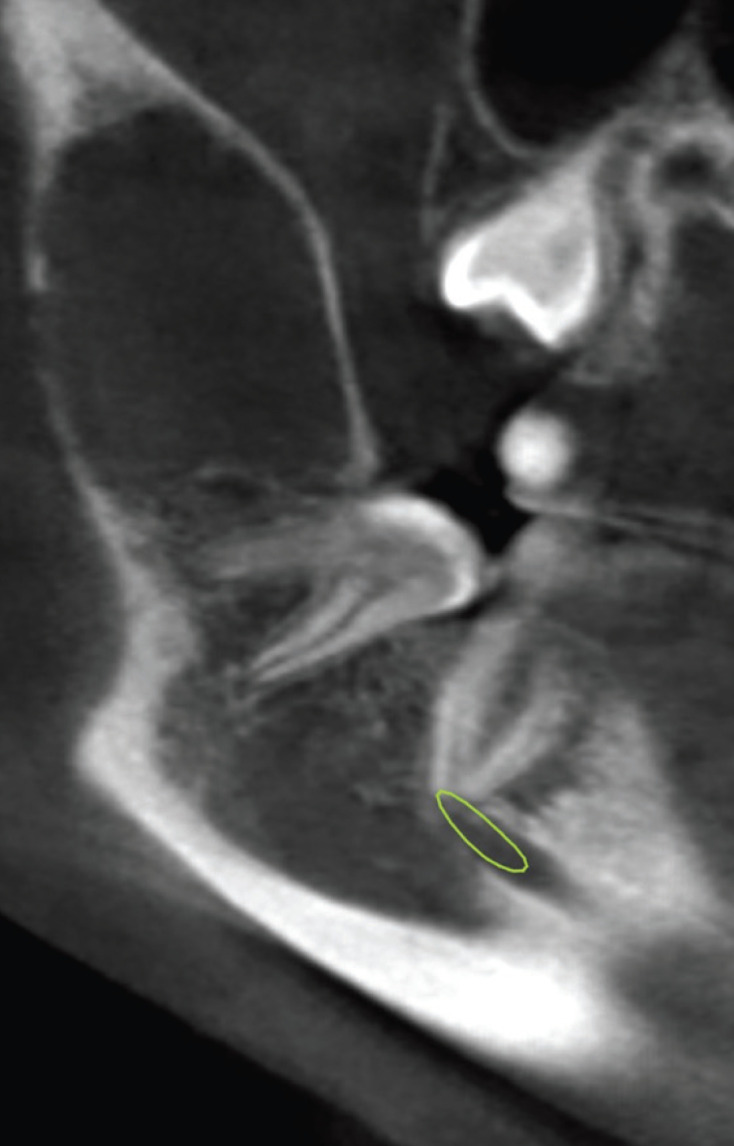
Cone-beam computed tomography of the simple bone cyst. Sagittal view.

**Figure 2:**
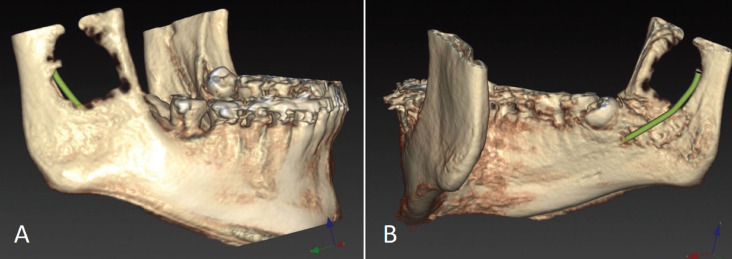
Cone-beam computed tomography of the simple bone cyst: 3D reconstruction. A) Buccal aspect, B) Lingual aspect.

A 16-year-old man with no relevant medical history attended the dental clinic referred by another clinician presenting a radiolucent image in the right ascending branch of the mandible, casually found on a control orthopantomography. The patient was asymptomtic and did not reported any history of trauma. A cone-beam computed tomography (CBCT) was made and showed a unilocular radiolucent lesion in the mandibular angle and the right ascending ramus of 3x2.5 centimeters, with smooth and ovoid edges, without calcifications or septa, which discreetly expanded the bone cortex (Figs. 1,2). The radiological differential diagnosis was odontogenic keratocyst and dentigerous cyst due to its proximity to the semi-included third molar.

After signing the informed consent, the surgery was performed under intravenous sedation (conscious sedation with propofol) and local anesthesia with 4% articaine (1:100.000 epinephrine) (Inibsa, Lliçà de Vall, Barcelona, Spain). First, the extraction of the semierupted 4.8 was made by performing a triangular mucoperiosteal flap and ostectomy/odontosection with a number 8 tungsten carbide drill connected to hand-piece under saline irrigation. Then, the distal crestal incision was extended to the retromolar area and a full thickness flap was elevated. Ostectomy was made until the lesion was reached. Intraoperatively, an empty cavity with the presence of some soft tissue at the bottom was found and the curettage of the lesion was made. The lesion and also some small samples of the bone wall were sent for the anatomopathological study. Finally, the wound was sutured with 3/0 silk (Ethicon, MersilkTMSoie, Edinburgh, UK) achieving a primary closure.

Postoperative medication was one Tablet of 750 mg amoxicillin plus 125 mg clavulanic acid (Augmentine®, Glaxosmithkline, Mayenne, France) every 8 hours since 2 days before until one week after the surgery, 4 mg dexamethasone (Fortecortin®, Laboratorios ERN, Barcelona, Spain) during 6 days in a descending pattern and one Tablet of 600 mg ibuprofen (Neobrufen®, Mylan Pharmaceuticals, Barcelona, Spain) every 8 hours during a week.

The anatomopathological study did not find histological signs of malignancy, tuberculoid granulomas, or any type of epithelium or multinucleated osteoclastoid cells. The analysis revealed bone fragments and connective-vascular remains with cholesterol, so the definitive diagnosis was simple bone cyst.

No postoperative complications appeared at the postoperative period neither at 6 months of follow-up. At this time, a new CBCT showed that the cavity was partially filled with bone.

## Discussion

This case report presents clinical and radiological characteristics that could be initially correlated with a dentigerous cyst or odontogenic keratocyst. However, the definitive diagnosis was SBC despite of its lower prevalence compared to the other entities (3,4). In general, SBCs are detected incidentally through the radiographic exam. The final diagnosis is only obtained through clinical and histological examination that detect an intraosseous cavity, empty or filled with serous or blood fluid (4).

In the reported case, the anatomic situation of the cyst and its possible relationship with the semierupted third molar required a differential diagnosis with the dentigerous cyst. Currently, the dentigerous cyst represents around 23% of all odontogenic cysts, generally appears in men in the third decade of life and mainly associated with impacted third molars, up to 45% of cases (8). After surgery, the possibility of a dentigerous cyst was ruled out, as it was not related to the semi-included third molar nor presented epithelium.

The first-line treatment is surgery along with curettage of the lesion walls as it promotes the formation of a blood clot, necessary for the subsequent bone repair (4,9). Other treatments such as marsupialization, marginal resection, or surgical access only has also been described. A higher rate of persistence after treatment was observed for surgical access only compared to curettage, and for multiple SBCs compared to solitary SBCs. Moreover, large or scalloping lesions around the teeth may hinder the formation of a blood clot suffcient for bone repair in some circumstances, which may explain the persistence or recurrence of the lesion (9). Regarding the addition of materials that promote bone regeneration, Trabizi *et al.* (10) published a single-blind randomized clinical trial which compared the use or not of protein-rich plasma for the treatment of SBCs and observed an improvement of bone formation compared to the curettage alone. Buchbender *et al.* (11) recommend use of autologous bone substitutes because of their regenerative properties, especially in cases with a defect greater than a critical size decfect with <1 cubic centimeter (cm3). To date there is no study comparing autologous bone with any kind of bone substitute material in a prospective comparative treatment approach. The current literature does not include any studies using xenogeneic or allogeneic subtitutes for the augmentation of cystic defects.

Although spontaneous resolution of untreated SBCs may occur (12), a recent review (9) recommends to avoid periodic observation of suspected SBCs without surgical intervention because it may continue to increase if left untreated and may eventually lead to pathological fractures of the affected bone. Furthermore, control visits after the surgical intervention must be done to ensure the new bone formation and reduction of the bone cavity.

## Conclusions

SBCs are usually discovered incidentally by a radiological study and surgical intervention is required to confirm the diagnosis and promote bone regeneration. Control visits are necessary to check the bone formation and confirm the absence of postoperative complications.
